# Use of airborne lidar data to improve plant species richness and diversity monitoring in lowland and mountain forests

**DOI:** 10.1371/journal.pone.0184524

**Published:** 2017-09-13

**Authors:** Marc Bouvier, Sylvie Durrieu, Frédéric Gosselin, Basile Herpigny

**Affiliations:** 1 Irstea, UMR TETIS, 500 rue Jean-François Breton, Montpellier, France; 2 Info Geo Drones, Pépinière d'Entreprises Versailles Grand Parc, Versailles, France; 3 UR EFNO, Irstea, Domaine des Barres, Nogent-sur-Vernisson, France; Chinese Academy of Forestry, CHINA

## Abstract

We explored the potential of airborne laser scanner (ALS) data to improve Bayesian models linking biodiversity indicators of the understory vegetation to environmental factors. Biodiversity was studied at plot level and models were built to investigate species abundance for the most abundant plants found on each study site, and for ecological group richness based on light preference. The usual abiotic explanatory factors related to climate, topography and soil properties were used in the models. ALS data, available for two contrasting study sites, were used to provide biotic factors related to forest structure, which was assumed to be a key driver of understory biodiversity. Several ALS variables were found to have significant effects on biodiversity indicators. However, the responses of biodiversity indicators to forest structure variables, as revealed by the Bayesian model outputs, were shown to be dependent on the abiotic environmental conditions characterizing the study areas. Lower responses were observed on the lowland site than on the mountainous site. In the latter, shade-tolerant and heliophilous species richness was impacted by vegetation structure indicators linked to light penetration through the canopy. However, to reveal the full effects of forest structure on biodiversity indicators, forest structure would need to be measured over much wider areas than the plot we assessed. It seems obvious that the forest structure surrounding the field plots can impact biodiversity indicators measured at plot level. Various scales were found to be relevant depending on: the biodiversity indicators that were modelled, and the ALS variable. Finally, our results underline the utility of lidar data in abundance and richness models to characterize forest structure with variables that are difficult to measure in the field, either due to their nature or to the size of the area they relate to.

## Introduction

Forest structure affects both microclimate and habitat quality and is therefore a key factor driving biodiversity in forest ecosystems [1]. Several studies have highlighted the existence of links between forest structure and wildlife richness [2–4] or floristic diversity [5,6], and understory vegetation is known to be particularly sensitive to forest structure [7]. However, what drives plant species distribution and composition over different forest habitats is still unclear [8].

The term “forest structure” refers to the spatial arrangement of the forest vegetation. Structure may be characterized based on four aspects: (i) diversity and mixture of species, (ii) horizontal and vertical heterogeneity, (iii) gap distribution; and (iv) coarse woody debris [9]. Vertical distribution of vegetation and dominant height are widely used variables in ecological modelling [10]. Simonson et al. [11] highlighted the importance of variables related to mean canopy height for plant species composition and diversity. Meanwhile, gaps in the under- and overstory have short-term impacts on biodiversity through different mechanisms [12]. Moreover, Duguid and Ashton [13] found that silvicultural systems based on small gaps favoured floristic biodiversity.

Establishing statistical models that can reliably describe the link between biodiversity and forest structure remains challenging. However, such models would facilitate the implementation of sustainable management strategies and practices. Therefore, methods that would improve our capacity to measure and describe three-dimensional vegetation structures should be developed. These would provide appropriate and reliable structural indicators that could be used as input variables for biodiversity models.

The Bayesian statistical approach is a highly promising framework when addressing biodiversity modelling issues. It can be used to draw inferences on large numbers of variables describing complex relationships, which are an intrinsic part to ecological studies [14]. As with other parametric statistical methods, Bayesian statistical models can provide an estimate of the magnitude of the relationship between biodiversity indicators and ecological variables. They offer great flexibility of use by allowing the integration of new probability distributions, which characterize ecological count data, and to shift to nonlinear models. Nevertheless, one of the main limitations of Bayesian models is that Bayesian inference cannot be solved using analytical approaches, and thus numerical solutions involve solving high-dimensional integration problems [15]. According to Wilkinson [15], the recent development of carefully crafted Markov chain Monte Carlo (MCMC) algorithms, in conjunction with increased computer speeds, has been a crucial step in overcoming this issue. Another limitation—shared with other parametric statistical methods—lies in the large quantity of calibration data needed to accurately estimate model parameters when the model contains several explanatory variables [16]. Zilliox and Gosselin [17] successfully studied the link between floristic biodiversity and both abiotic and biotic environmental variables following a Bayesian approach they had developed. The authors found that forest structure variables had a non-negligible relationship with species richness for selected floristic ecological groups, and that this effect varied among ecological groups and according to ecological conditions.

Forest structure is commonly measured using traditional field measurements, as is the case in most biodiversity studies. However, some structural indicators that are relevant for biodiversity studies are difficult to assess using ground surveys [18,19]. To overcome these limitations, remote sensing is increasingly used as an alternative to field surveys as it provides quick and accurate structure measurements over large areas, including metrics which are difficult to measure from the ground (e.g. height measurements). For example, Getzin et al. [7] showed that gap distribution is a major driver of understory plant diversity in deciduous forests using high-resolution aerial images. Nevertheless, optical sensors do not provide the three-dimensional (3D) information needed to characterize forest structures [20]. To this end, the potential of lidar (Light detection and ranging), which is a technology based on the emission and reception of laser pulses, has been widely acknowledged [21,22], in particular that of airborne laser scanner (ALS). Lidar technology provides an opportunity to build variables describing aspects of the forest structure which differ from those observed, or measured, during field surveys. These new variables may be more suitable for describing the link between forest structure and understory vegetation.

ALS data have been used to analyse relationships between biodiversity indicators and a range of structural variables related to the three-dimensional arrangement of vegetation [18,19,23–26]. For example, Simonson et al. [11] used ALS data to model plant species composition and diversity in Mediterranean oak forests. They found that ALS-measured vegetation height was positively associated with species diversity. Lopatin et al. [27] also used a partial least squares path model (PLS-PM) algorithm based on ALS data to predict plant richness from variables related to topography, and to both crown cover and tree height in Mediterranean forests. Complementing ALS variables with environmental variables has improved the predictive power of floristic biodiversity models [28,29]. To move further in this direction would entail using additional non-biotic variables and a modelling framework that can better address the complexity of biodiversity models.

Biodiversity modelling could therefore be improved by combining the significant advances in Bayesian modelling and input variables enriched with ALS data that would provide more appropriate and reliable structure measurements. The aim of our study was to demonstrate the utility of ALS variables when attempting to highlight and better understand the influence of forest structure on understory biodiversity. Floristic biodiversity was assessed based on the species abundance and richness of three ecological plant groups. ALS variables were derived as structural measurements to describe certain characteristics resulting from the spatial arrangement of trees in a stand. Two specific objectives were identified: (1) to evaluate if ALS variables linked to vegetation characteristics assumed to be potential drivers of understory biodiversity in the existing literature (e.g. tree height, gaps, canopy volume) did indeed improve Bayesian biodiversity models; and (2) to determine if the capacity to predict the effect of forest structure on local biodiversity is dependent on the size of the area considered for structural measurements around the floristic survey plots. If so, this would reinforce the assumption that local floristic biodiversity of the understory is influenced by the neighbouring structural characteristics of a stand [30], which cannot be demonstrated using traditional field data.

## Materials and field data preparation

### Study sites

Two forested areas in North-eastern France, which are partially covered by ALS surveys, were selected for their differences in terms of topography and tree species diversity: a lowland forest comprised of multi-layered deciduous stands (Lowland site), and a mountain forest comprised of coniferous, deciduous and mixed stands (Mountain site). The Lowland site is a 10,000 km^2^ area located in the Lorraine region (48.53° N, 5.37° E). The regional climate is semi-continental and subject to an oceanic influence [31]. The Lorraine lowland forest is fragmented and intensively managed. In the selected area, forests are dominated by European beech (*Fagus sylvatica* L.), European hornbeam (*Carpinus betulus* L.) and Sycamore maple (*Acer pseudoplatanus* L.). The Mountain site is a 9,340 km^2^ area located in the Vosges region (48.03° N, 7.08° E). The regional climate is semi-continental [31]. The area is characterized by hilly topography, with elevations ranging from about 120 m to 1420 m. The stands are typically heterogeneous and uneven-aged, and are dominated by European beech, European silver fir (*Abies alba* Mill.) and Norway spruce (*Picea abies* (L.) H.Karst).

### ALS data

Data were collected at both sites using small-footprint ALSs. Only a partial sub-area of each site was covered by the ALS survey. Specifications on the ALS data are given in Table 1.

**Table 1:** Technical specifications for the ALS data that were acquired and summary of field variables for both study sites.

	Lowland site	Mountain site
Sub-area (km^2^)	60	1,200
Date of survey	October 2010	March and April 2011
ALS sensor	LMS–Q560—Riegl (Austria)	ALTM 3100—Optech (Canada)
Wavelength (nm)	1550	1064
Scan angle (°)	29.5	16
Pulse density (pulses/m2)	20.7	3.4
Flight altitude (m a.g.l.)	550	1,500
*Reaction(pH)*	5.2 ± 0.6 [3.5; 6.7]	4.1 ± 0.5 [2.9; 6.3]
*SWC*	5.1 ± 0.3 [4.5; 6.9]	5.2 ± 0.3 [4.4; 7.7]
*T*_*mean*_ (°C)	9.3 ± 0.4 [8.7; 10.5]	8.6 ± 0.7 [6.2; 10.2]
*Solrad* (MJ/m^2^/day)	639.3 ± 22.5 [563.1; 682.5]	614.9 ± 39.8 [479.3; 710.1]
*Elevation* (m)	300.7 ± 79.5 [108.0; 488.0]	524.7 ± 224.0 [120.3; 1,419.8]
*Slope* (%)	9.6 ± 7.4 [0.0; 29.4]	27.1 ± 18.8 [0.0; 85.0]
*Aspect* (grades)	208.3 ± 123.2 [0.0; 400.0]	210.6 ± 114.0 [0.0; 400.0]
*C*_*tot*_ (%)	76.7 ± 29.7 [0.0; 150.0]	83.5 ± 24.1 [2.5; 170.0]

Data pre-processing was performed for each study area by the data providers: Sintegra (France) and the French National Institute of Geographic and Forest Information (IGN, France) for the Riegl and Optech data, respectively. Ground points were classified following the TIN-iterative algorithm [32] in order to produce a digital terrain model (DTM). Next, first return points were extracted from the data to produce a digital surface model (DSM). Both the DTM and DSM had a 1 m resolution. For each acquisition, aboveground heights were calculated by subtracting the ground elevation given by the DTM from each corresponding ALS elevation point; thereby removing topographic effects from the ALS point clouds. From the resulting ALS point clouds, four sub-point clouds were extracted for each field plot; these point subsets corresponded to various spatial extents (plot radius; and 50 m, 100 m, and 200 m around the study plots) where local biodiversity was assessed in the field.

### Field inventories

Field data were collected on 789 circular plots within the Lowland site. For the 48 plots (9m radius) within the sub-area covered by ALS data, field data were obtained from the EcoPlant database [33], and for the remaining 741 plots (15 m radius), data came from the IGN database (http://inventaire-forestier.ign.fr/spip/spip.php?article707). At the Mountain site, data were obtained from the IGN for 1,155 circular plots. 171 (15 m radius) were surveyed within the sub-area covered by ALS data, and the remaining 984 (15 m radius) plots were located outside the sub-area. At both study sites, field data were collected from 2008 to 2012. Field plots covered in ice and snow during the inventories were excluded from the dataset. Plot centre positions were measured using a differential global positioning system (DGPS).

Soil characteristics, i.e soil pH (*Reaction*) and soil water capacity (*SWC*), were derived from the mean Ellenberg values of the understory species at both sites. Temperatures (*T*_*mean*_; °C) and global solar radiation (*Solrad*; MJ/m^2^/day) were based on May to September average values. Monthly values were obtained from the French National Meteorological Service (Météo-France). *Solrad* was estimated from temperature data using the equation in [34]. The spatial resolution of the meteorological data was 1 km^2^. Topography was described by three variables, i.e. Type of Topographical Situation *(TTS)*, Slope, and Aspect. *TTS* was defined according to the French National Forest Inventory documentation as follows: 0—flat terrain; 1—summit (sharp, round or escarpment); 2—top part of a slope; 3—concave mid-slope; 4—straight mid-slope; 5—convex mid-slope; 6—flat profile on a slope; 7—bottom part of the slope; 8—wide valley; and 9—closed depression (see Figure SM.1 in Appendix A. in Zilliox and Gosselin [17]). Flat topographies (0, 6, and 8) were not distinguished in the dataset. *Aspect* was defined as the magnetic azimuth (grades) of the steepest slope of the plot.

Total tree crown cover (*C*_*tot*_; %) was estimated at both study sites from field inventories. *C*_*tot*_ is the ratio between the projected surface area of all individual tree crowns and the total plot surface area. In multi-layered stands, *C*_*tot*_ can exceed 100%. *C*_*tot*_ was chosen as an indicator of light penetration through the vegetation cover. *C*_*tot*_ is considered to be a relevant synoptic biotic factor for biodiversity studies of understory plants [35–37].

Understory species were identified and their abundance estimated for each field plot and a Braun-Blanquet cover class [38] was attributed to each of the eight most abundant species in each study region (Table 2). The Braun-Blanquet cover classes that we used distinguish 6 cover classes ranging from 0 to 5 depending on the cover percentage of each species in the plot (absence, less than 5%, between 5 and 25%, between 25 and 50%, between 50 and 75%, more than 75% cover). Species richness was also estimated for functional groups of species based on light preference. Three species classes were distinguished based on Ellenberg values 1 to 9: shade-tolerant, from 1 to 3 (shade); intermediate-light, from 4 to 6 (mid); and heliophilous species, from 7 to 9 (helio) [39]. The Ellenberg value is an indicator of the tolerance of a given species to several environmental parameters. These values were used to scale the flora at the two study sites along gradients reflecting light, temperature, soil pH, fertility, continentality, moisture, and salinity levels. The Julve [40] autecological table of correspondence was used to assess the Ellenberg values of each species.

**Table 2:** List of the eight most abundant species at each study site. Species are ranked in order of decreasing abundance.

Lowland site			Mountain site		
Species name	Species code	Ellenberg value	Species name	Species code	Ellenberg value
*Brachypodium sylvaticum* (Huds.) P.Beauv.	brsy	4	*Carex pilulifera* L.	Capi	5
*Carex sylvatica* Huds.	casy	5	*Deschampsia flexuosa* L.	Defl	8
*Galium odoratum* (L.) Scop	gaod	3	*Hedera helix* L.	Hehe	3
*Hedera helix* L.	hehe	3	*Oxalis acetosella* L.	oxac	4
*Lamium galeobdolon* (L.) L.	laga	4	*Rubus idaeus* L.	ruid	5
*Milium effusum* L.	mief	5	*Vaccinium myrtillus* L.	vamy	5
*Anemone nemorosa* L.	anne	4	*Digitalis purpurea* L.	dipu	5
*Poa nemoralis* L.	pone	7	*Athyrium filix-femina* (L.) Roth	atfi	3

## Methods

In our study, we focused on ground-layer floristic abundance and richness. We considered abundance for the eight most representative species at each study site, excluding woody species. We also considered species richness for three ecological groups based on light preference, i.e. shade-tolerant, mid-light preferring, and heliophilous. We developed Bayesian models to link species richness and abundance with both environmental and ALS variables. In accordance with Austin and Van Niel [28], we included seven environmental abiotic variables in our models: *Reaction*, *SWC*, *T*_*mean*_, *Solrad*, *TTS*, *Slope* and *Aspect*, as well as one tree stand variable, *C*_*tot*_, as an indicator of forest structure. As in Austin and Van Niel [28], we included a single biotic variable. Indeed, using Bayesian models requires a high ratio between reference data and explanative factors to provide reliable and interpretable results. Therefore, the limited size of the sub-areas covered by ALS data in our study, and the resulting size of the two reference data sets, compelled us to use only one biotic variable in the models. Data processing was performed in the R statistical environment version 3.1.1 (http://www.r-project.org/).

### ALS variables

Several ALS variables were identified and used as descriptors of the 3D vegetation structure in floristic statistical models (Table 3). The variables were extracted from circular plots with the same radius as the field plots (9 m at the Lowland site and 15 m at the Mountain site respectively), and also for three other scales with 50 m, 100 m and 200 m radii. For the sake of parsimony when selecting possible explanative factors, and in order to compare the variables with each other, the ALS variables were tested individually. They were then used to assess both the magnitude and direction of the relationship between biodiversity indicators and the forest structure components characterizing the plot itself or its wider surrounding environment. Variables were related to vegetation height characteristics (H_max_, *H*_*median*_, *H*_*mean*_), vertical heterogeneity ($\sigma_{H}^{2}$, *Gini*, *Cv*_*LAD*_), horizontal canopy cover (*Gap*_*max*_, *C*_*f*_, *C*_*r*_) and both vertical and horizontal canopy development (*Vol*_*can*_).

**Table 3:** Description and summary of forest structure variables derived from ALS data. With *z*_*i*_ corresponding to the aboveground height of an ALS point *i*, *n* to the total number of ALS points, and *N* to the total number of 1 m^2^ grid cells in the plot. Variables were extracted from circular plots with the same radius as the field plots (9 m at the Lowland site (L) and 15 m at the Mountain site (M) respectively). Vegetation points inferior to 2 m were considered to belong to the understory and were not taken into account as tree vegetation points when computing the following variables: *H*_*mean*_, $\sigma_{H}^{2}$, *Gini*, *Cv*_*LAD*_, *Gap*_*max*_, *C*_*f*_, *C*_*r*_.

ALS variable	Variable description	Site	μ±σ *[min;max]*
*H*_*max*_ = *max(z*_*i*_*)*	Maximum point height	L	21.68 ± 5.99 *[7*.*17; 40*.*66]*
		M	26.77 ± 7.62 *[5*.*86; 48*.*40]*
*H*_*median*_ = *median(z*_*i*_*)*	Median point height (all points, including ground points)	L	13.5 ± 5.79 *[2*.*82; 34*.*73]*
		M	11.72 ± 7.93 *[1*.*12; 29*.*4]*
$H_{mean}=\frac{1}{n}\;\sum_{1}^{n}z_{i}$	Mean point height above 2 m.	L	14. ± 5.24 *[4*.*01; 34*.*43]*
		M	17.57 ± 6.41 *[3*.*06; 33*.*66]*
$\sigma_{H}^{2}=\;\frac{1}{n}\sum_{1}^{n}{\left(z_{i}-H_{mean}\right)}^{2}$	Variance of point height above 2 m.	L	15.56 ± 17.23 *[0*.*98; 109*.*88]*
		M	24.67 ± 1.03 *[0*.*56; 126*.*60]*
$Gini=\;\frac{\sum_{1}^{n}\left(2i-n-1\right)\;z_{i}}{\sum_{1}^{n}z_{i}\left(n-1\right)}$	Gini coefficient above 2 m [41]. *Gini* has a theoretical minimum value of zero, expressing perfect equality when all ALS points are of the same height value; it takes a theoretical maximum value of one, indicating greater diversity when all ALS points except one have a height value of zero.	L	0.23 ± 0.12 *[0*.*09; 0*.*67]*
		M	0.47 ± 0.18 *[0*.*10; 0*.*82]*
*Cv*_*LAD*_	The coefficient of variation in leaf area density above 2 m was calculated as the ratio of the standard deviation to the mean of the leaf area density (*LAD*)^(1)^ profile [42].	L	0.96 ± 0.3 *[0*.*53; 2*.*41]*
		M	1.29 ± 0.95 *[0*.*72; 10*.*11]*
*Gap*_*max*_	Maximum gap size above 2 m was computed from the canopy height model (DSM-DTM) using the clump function from the raster package in the R software.	L	12.12 ± 29.61 *[0; 157]*
		M	70.22 ± 113 *[0; 674]*
$C_{f}=\;\frac{N_{\left(DSM-DTM\right)>2m}}{N}$	The cover fraction above 2 m was defined as the proportion of vegetation cover over total plot area.	L	0.97 ± 0.05 *[0*.*72; 1]*
		M	0.84 ± 0.17 *[0*.*06; 1]*
$C_{r}=\;\frac{n_{z>2m}}{n}$	Attenuation rate above 2 m; *C*_*r*_ is related to light penetration through the canopy cover [44]. Unlike *C*_*f*_, *C*_*r*_ takes into account gaps smaller than 1 m^2^.	L	0.94 ± 0.09 *[0*.*57; 1]*
		M	0.8 ± 0.2 *[0*.*03; 1]*
$Vol_{can}=\sum_{1}^{N}\left(DSM-DTM\right)$	The total canopy volume was defined as the volume between the DSM and the DTM [45].	L	11483 ± 4141 *[3295; 27065]*
		M	11263 ± 4745 *[371; 21664]*

^(1)^ The *LAD* profile was computed by assessing a transmittance profile and then using the Beer-Lambert law to retrieve vegetation density at each height interval (*dz*): $LAD=\;-\frac{\text{ln}\left(Pen_{r}\right)}{k\;dz}$, with k the extinction coefficient approximated by 0.5 [43]

### Statistical models

Our statistical models were Bayesian and included probability distributions of statistical parameters prior to data observation called prior distributions. These were updated to probability distributions after data observation called posterior distributions [46].

**Fig 1:**
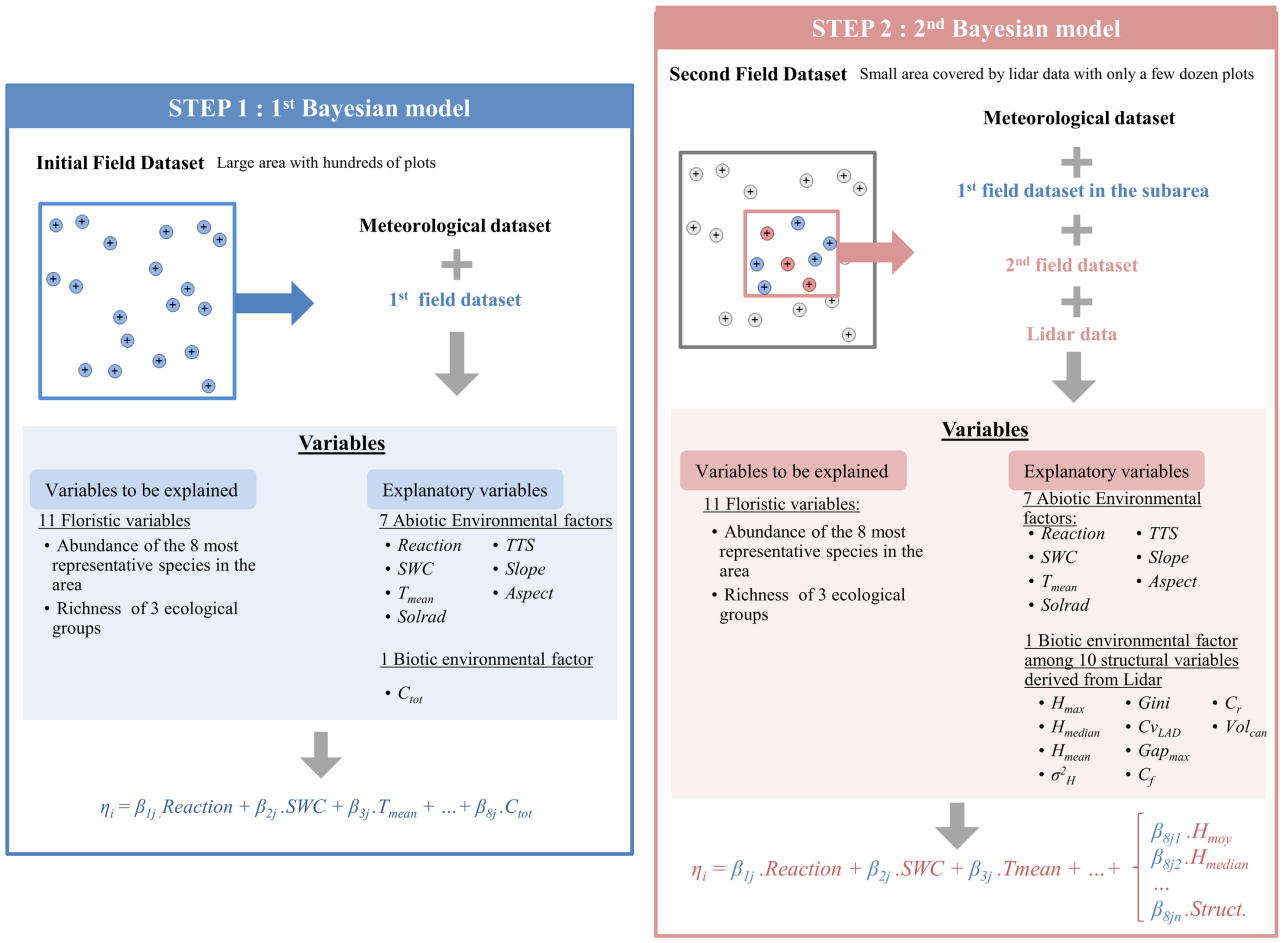
Process diagram describing the modelling framework developed to link species richness and abundance with both environmental and ALS variables. For the sake of clarity, the model presented in this diagram is a simplified shape of the real model, presented in Appendix. Analyses were carried out on the results from the second step.

As ALS data did not cover the whole area for both sites, and only incorporated a limited number of plots (48/789 for the Lowland site and 171/1,155 for the Mountain site), we used a two-step approach to build our models (Fig 1). The first step aimed at estimating model parameters for all the plots at each of the sites with the cover rate *C*_*tot*_ as the stand biotic variable since it was the only explanatory biotic variable collected in both areas under consideration. In a second step, models were fitted on a smaller sample of field plots within the ALS sub-area, based on the posterior probability functions computed in the first step for abiotic variables.

The statistical models that were used had the same structure as the models in Herpigny and Gosselin [47] for species abundance and in Zilliox and Gosselin [17] for species richness. The abiotic variables are those given in section 2.3, along with the categorical variable TTS (Type of Topographical Situation) which was converted into a numerical variable.

The probability distributions of the observed data were respectively the Bernoulli/Double Polya mixture-Poisson-Negative Binomial family for species richness—which allows for both under- and over-dispersion relative to the Poisson distribution [17]–and the MTUnlimited 2 zero-inflated cumulative beta distribution for abundance data.

In the second step, our models only included *Reaction* and *SWC* as abiotic variables, with the same shape as above, but here, the parameters of these variables were held fixed at their mean posterior density distribution estimated in the first step. Indeed, using the full distribution set would have required integrating the distribution in our MCMCs, which would probably have significantly slowed down our estimations. In this second step, the biotic variable *C*_*tot*_ was replaced, in turn, by each one of the ALS variables (cf. Table 3) in order to evaluate the individual contribution of each ALS variable to the model. The β parameter associated with this variable, the intercept and the “nuisance” parameters (e.g. the Index of Dispersion for count data models) were the only ones estimated in the statistical models in this second step since the parameters corresponding to the seven abiotic variables were taken from their posterior distribution in the models from the first step (cf. above).

The Bayesian models were fitted through an adaptive Markov-Chain Monte Carlo (MCMC) programmed in R and C, calling some C functions that one of the members of our research team (FG) coded. This MCMC was inspired by Gregory [48]. The main changes made to the process proposed by Gregory [48] and the convergence conditions of the MCMC are summarized in Appendix. Once convergence was reached, we simulated 2,000 values of the parameters.

### Statistical analyses

Our first indicator of the statistical models was a Deviance Information Criterion (DIC) calculated for each model. As recommended by Richardson et al. [49], we used the mode-based DIC to compare Bayesian models. We assessed the difference in *DIC* (*ΔDIC*) between models with and without an ALS predictor. The lower the *ΔDIC*, the better the model, and the greater the improvement brought about by using the ALS variable.

In addition, we assessed the statistical significance, magnitude and direction of the effects of ALS variables on species abundance and richness for each model [17,50]. For significance, we estimated through empirical quantiles the two-tailed significance test of the difference between 0 and the statistical parameter β associated to the ALS variable. *p*-values were classified between bounds 0, 0.01, and 0.05, thereby yielding three intervals. We considered *p* ≤ 0.01 (symbolized by **) highly significant; 0.01 < *p* ≤ 0.05 (*) significant; and 0.05 < *p* non-significant. Magnitude and direction of the effects of ALS variables were evaluated in order to assess the impact of the ALS variables. Our approach consisted in studying the effect on the mean of the biodiversity variable of an increase of one standard deviation *sd* for the selected ALS variables associated to each model parameter β. Equivalence testing was used to detect the negligible effects of a given ALS variable on the model [51,52]. This test enabled us to identify cases where the effect of the one *sd* increase in the ALS variable on the logit (for abundance) or logarithm (for species richness) of the mean of the biodiversity indicator had an empirical probability above 0.95 of being within an interval that corresponded either to: negligible, non-negligible positive or non-negligible negative effects. This allowed us to distinguish: (i) cases where the effects were estimated as weak, (ii) cases where the effects were strong and positive, (iii) cases where the effects were strong and negative, or (iv) cases where the estimators were too noisy to conclude. More technically, we denoted the levels associated to negligible intervals as b_1_ and b_2_, with b_1_ = 0.25 and b_2_ = 0.5 for species abundance, and b_1_ = 0.1 and b_2_ = 0.2 for species richness of the three ecological groups. We therefore defined: (1) weakly negligible effects when the parameter had a high probability of being in the larger negligible interval, i.e. P(−b_2_<(β*sd)<b_2_) > 0.95 (symbolized by 0), and strongly negligible effects when the parameter had a very high probability of being in the narrower negligible interval, i.e. P(−b_1_<(β*sd)<b_1_) > 0.95 (symbolized by 00); (2) non-negligible negative and strongly non-negligible negative effects (symbolized by − and − −, respectively) when the effect of the variable increased by 1 *sd* had a 95% probability of being below −b_1_ and below −b_2_, respectively; and (3) non-negligible positive and strongly non-negligible positive effects (symbolized by + and ++, respectively) when the effect of the variable increased by 1 *sd* had a 95% probability of being above b_1_ and above b_2_, respectively.

### Analysis of statistical indicators

We investigated the influence of several ALS variables on predicting abundance for the eight selected species in each site and richness for the shade, mid, and helio groups. Since our main goal was to analyse the overall trend, we did not control for multiple comparisons. We observed the overall model improvement obtained with structural variables derived from ALS data; we identified the best ALS explanatory variables; and we explored the impact on model quality when the neighbouring surface area was included in vegetation structure characterization.

Firstly, we examined the count of models per class of effect, i.e. the combination of both magnitude and direction. We distinguished two types of magnitudes: significant and non-significant. For significant effects, we distinguished four types of directions: negligible, negative and positive, and no information (i.e. information was insufficient to draw reliable conclusions as to the magnitude of the effect for the studied variable). Only two types of directions were distinguished among non-significant effects: negligible effects, and insufficient information to distinguish between negligible and non-negligible effects. Secondly, we analysed the *ΔDIC* distributions obtained for each biodiversity indicator from the 40 models built with the different ALS variables. The models most improved by the use of an ALS variable were identified.

We analysed the *ΔDIC* distributions obtained for each ALS variable by considering: the 44 models built for the eleven biodiversity indicators, and the four neighbouring surface areas combined. For each ALS variable, the number of models for each level of significance and negligibility was also determined.

We compared the effects of the ALS variables on biodiversity models depending on the radius for the four different radii used to compute the ALS variables, i.e. the same radius as the field plots (9 m at the Lowland site and 15 m at the Mountain site), 50 m, 100 m and 200 m. Consequently, the number of ALS variables with significant or non-negligible effects—i.e. including those which were either negligible or which provided no information on negligibility—was identified for each radius. Then it was modelled as a function of the radius used to calculate the ALS variable with a binomial generalized linear model. The ALS variables were first considered as two-level variables: 9-15m vs the three other radii; and thereafter as four-level categorical variables. The local scale (9-15m) was first compared to the three other scales, then all four levels were compared with a Tukey multiple comparison procedure (function *glht* in the *multcomp* library).

## Results

The complete results for Lowland and Mountain sites are reported in S1 and S2 Tables, respectively.

### Overall analysis of model improvement with a structural variable from ALS

The level of significance and both the magnitude and the direction of improvement are summarized according to different effect classes in Table 4. The number of models per effect class differed depending on the study site. In the Lowland site, most of the ALS variables used in abundance models were found to be non-significant or with no information on negligibility (223/320). In contrast, most of the ALS variables used in richness models were found to be both non-significant and negligible (97/120), thus revealing the low potential of the selected ALS variables to improve the models on that site for most of the ecological groups. In the Mountain site, most of the ALS variables used in both the abundance and richness models were also found to be both non-significant and negligible (159/320 and 55/120, for abundance and richness respectively). However, some significant effects were observed, particularly for the Mountain site when compared to the Lowland site (120/440 and 36/440, respectively). It is worth noting that 27/440 of the ALS variables used in both abundance and richness models had significant negative effects or positive non-negligible effects in the Mountain site versus only 3/440 in the Lowland site.

**Table 4:** Number of abundance and richness models corresponding to each level of significance and negligibility of ALS variables at the Lowland and Mountain sites.

Effect class		Lowland site		Mountain site	
		Abundance	Richness	Abundance	Richness
Significant	Negligible	0	0	0	5
	Negative non-negligible	3	0	0	4
	Positive non-negligible	0	0	22	1
	No info	19	14	59	29
Non-significant	Negligible	75	97	159	55
	No info	223	9	80	26
Total models		320	120	320	120

**Fig 2:**
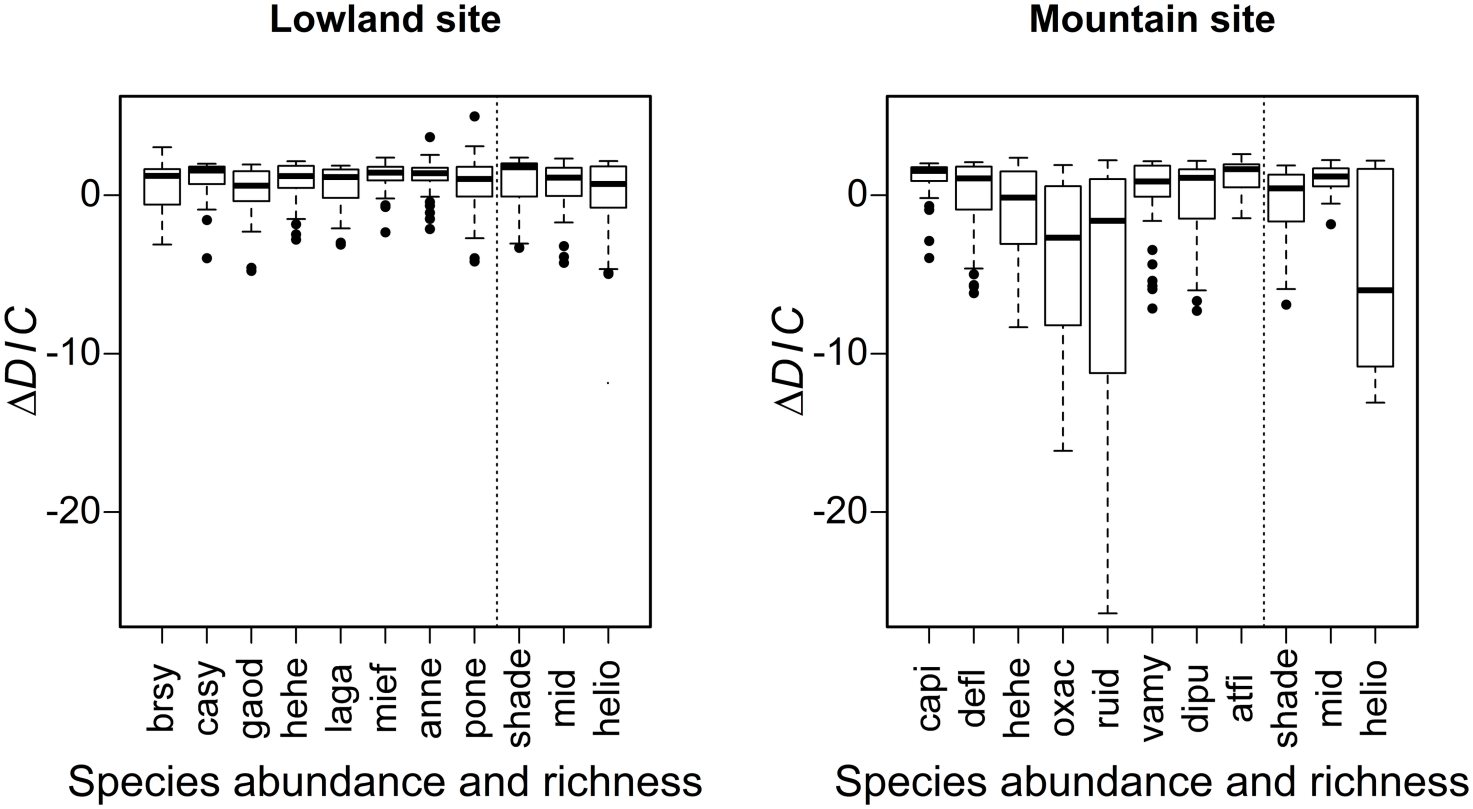
*ΔDIC* for floristic models depending on abundance and richness indicators in the Lowland and Mountain sites. Dark horizontal lines represent the median; boxes represent the 25th and 75th percentiles; whiskers the 5th and 95th percentiles; outliers are represented by dots. The lower the *ΔDIC*, the more the model is improved by the ALS variable.

Comparing *ΔDIC* helped to identify which species or ecological group models were most improved by an ALS variable (Fig 2), with lower *ΔDIC* corresponding to better models. In the Lowland site, the strongest improvement among species abundance and richness models was observed for heliophilous species richness with a minimum *ΔDIC* = -4.97. For all abundance and richness indicators, at least one ALS variable improved prediction. However, all median *ΔDIC* values were positive, i.e. ranging from 0.60 to 1.76. Median *ΔDIC* values were, on average, lower in the Mountain site than in the Lowland site: -0.24 and 1.20, respectively. In the Mountain site, raspberry bush abundance models showed the strongest improvement with a minimum *ΔDIC* = -26.38. A lower median *ΔDIC* value (-6.00) was found for heliophilous species richness.

### Identification of the best ALS explanatory variables

**Fig 3:**
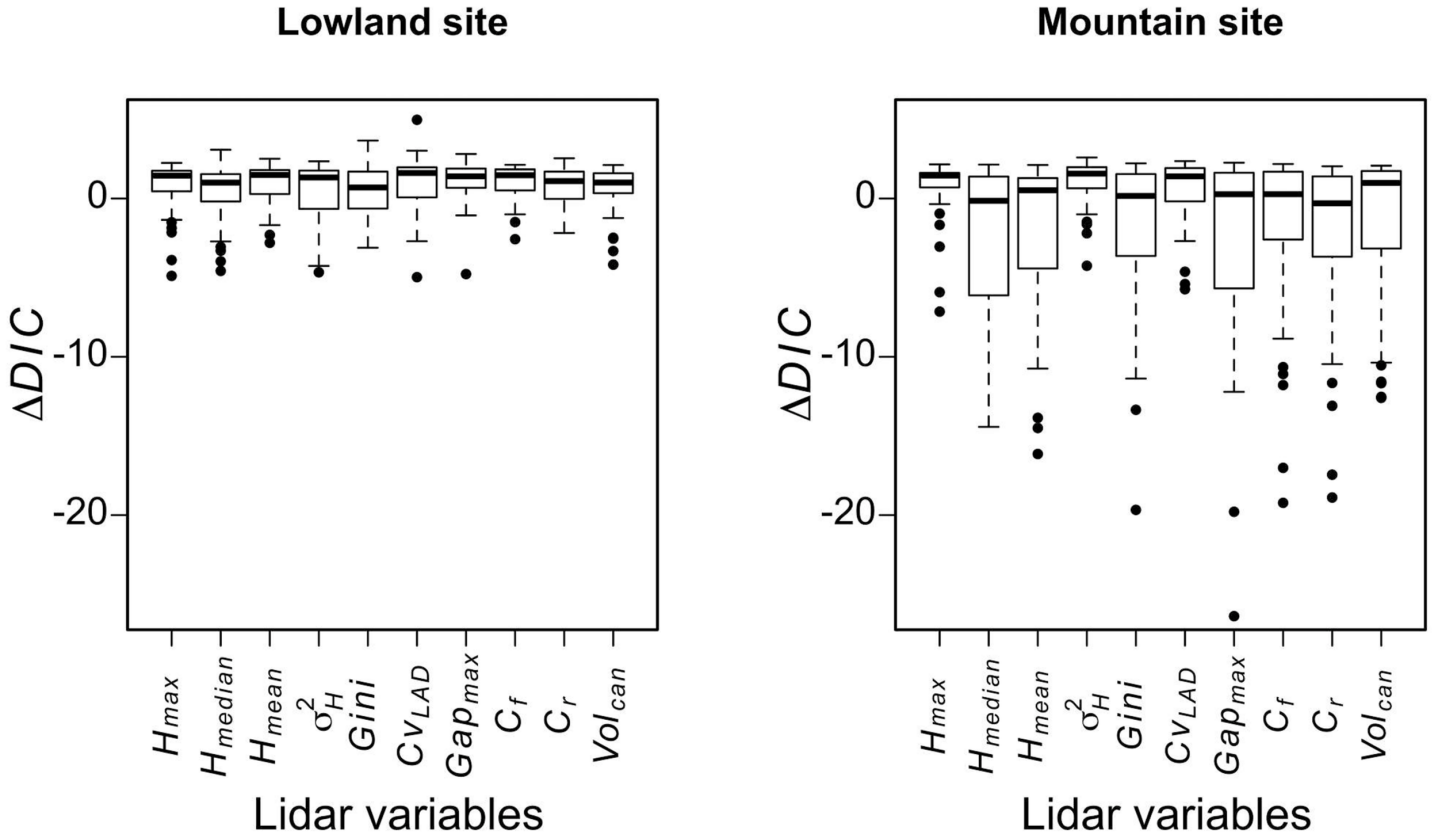
*ΔDIC* for abundance and richness models depending on ALS variables in the Lowland and Mountain sites. Dark horizontal lines represent the median; boxes represent the 25th and 75th percentiles; whiskers the 5th and 95th percentiles; outliers are represented by dots. The lower the *ΔDIC*, the more the model is improved by the ALS variable.

Comparison of *ΔDIC* also helped to identify the ALS variables that most improved biodiversity models irrespective of the indicators when also considering the four neighbouring surface areas (Fig 3). In the Lowland site, the greatest improvement was obtained using the *Cv*_*LAD*_ variable with a minimum *ΔDIC* = -4.97. The lowest median *ΔDIC* was found for the *Gini* variable (0.70). Overall, all ten variables improved model predictions for at least one model, with minimum *ΔDIC* values ranging from -4.97 to -2.18. However, only *H*_*max*_ had a significant non-negligible effect (negative), and this was true for three models (Table 5). On the Mountain site, the strongest improvement was obtained using a *Gap*_*max*_ variable with a minimum *ΔDIC* = -26.38. The lowest median *ΔDIC* (-0.30) was found for the *C*_*r*_ variable. All ten variables improved model predictions, with minimum *ΔDIC* values ranging from -26.38 to -4.25. However, *H*_*max*_, $\sigma_{H}^{2}$, and *Cv*_*LAD*_ appeared to be generally less explanatory than the other variables. This trend was confirmed by the analysis of both significance and magnitude of the effects. The three variables demonstrated non-significant effects in more models than did the others (respectively 41, 41 and 37 out of 44, versus fewer than 31 out of 44 for the other variables). Furthermore, they had a significant and non-negligible effect in 2, 0 and 1 models respectively, while all the other variables had a significant and non-negligible effect in at least 3 models (Table 5).

**Table 5:** Number of ALS variables corresponding to each level of significance and negligibility for abundance and richness models at the Lowland and Mountain sites.

Site	Effect class		*H*_*max*_	*H*_*median*_	*H*_*mean*_	$\sigma_{H}^{2}$	*Gini*	*Cv*_*LAD*_	*Gap*_*max*_	*C*_*f*_	*C*_*r*_	*Vol*_*can*_
Lowland site	Significant	*Negligible*	0	0	0	0	0	0	0	0	0	0
		*Negative non-negligible*	3	0	0	0	0	0	0	0	0	0
		*Positive non-negligible*	0	0	0	0	0	0	0	0	0	0
		*No info*	6	6	7	0	1	4	1	1	1	6
	Non-significant	*Negligible*	9	14	16	26	21	10	20	18	24	14
		*No info*	26	24	21	18	22	30	23	25	19	24
	Total models		44	44	44	44	44	44	44	44	44	44
Mountain site	Significant	*Negligible*	0	0	0	0	1	0	2	1	1	0
		*Negative non-negligible*	0	1	0	0	0	0	2	0	0	1
		*Positive non-negligible*	2	2	3	0	3	1	3	3	4	2
		*No info*	1	13	12	3	10	6	12	19	12	10
	Non-significant	*Negligible*	22	16	17	30	23	23	22	24	21	16
		*No info*	19	12	12	11	7	14	3	7	6	15
	Total models		44	44	44	44	44	44	44	44	44	44

### Impact on parameter estimation and model improvement of neighbouring vegetation structure

**Fig 4:**
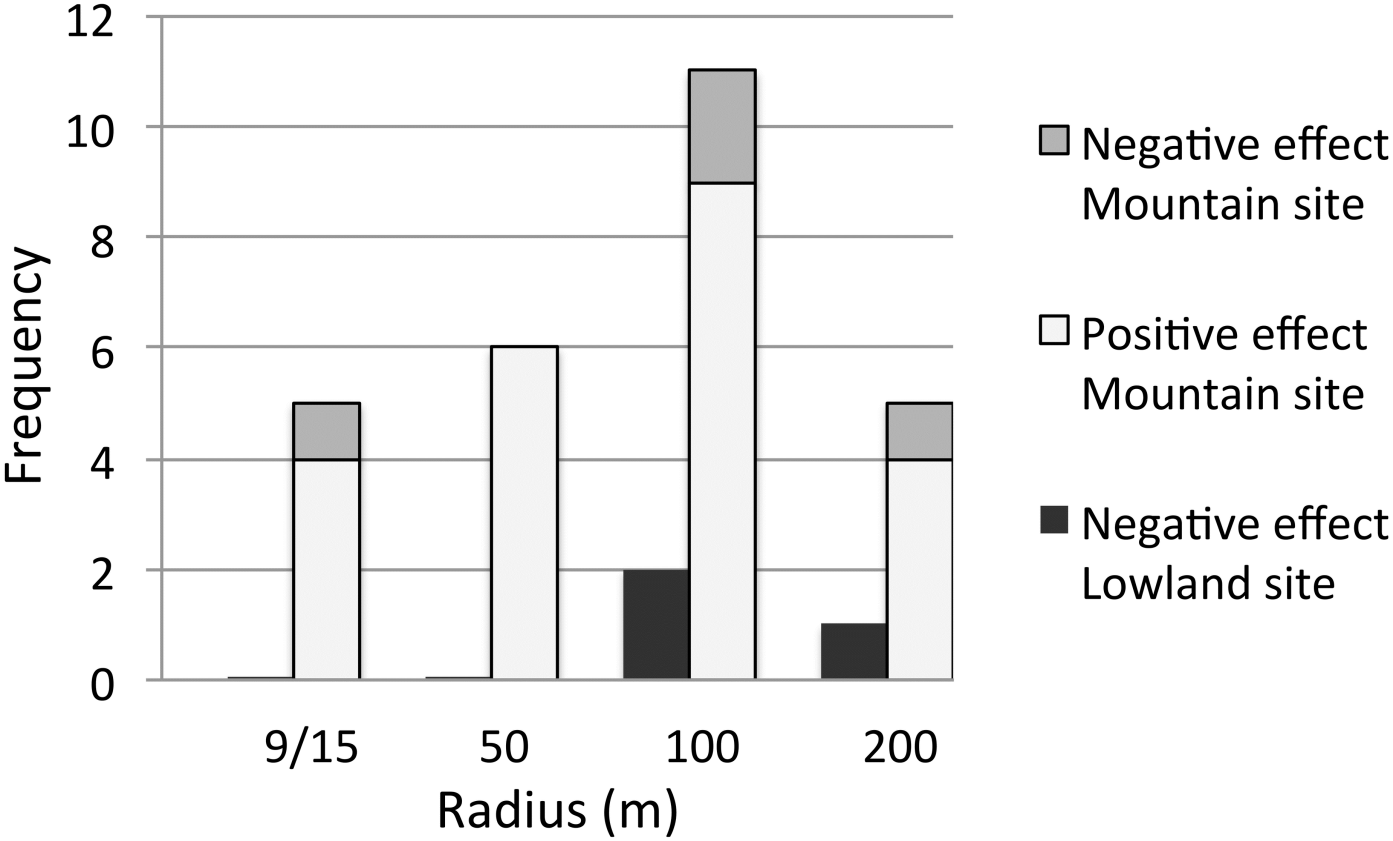
Number of ALS variables which were negative non-negligible or positive non-negligible when used in floristic models. Abundance and richness models were considered in both the Lowland and Mountain sites. The ALS variables were extracted from circular plots within the same radius as the field plots (9 m at the Lowland site and 15 m at the Mountain site), and also with radii of 50 m, 100 m and 200 m.

The proportion of ALS variables found to be statistically significant or non-negligible did not vary much with the radius of the circular plots used for extraction (Fig 4). In the Mountain site, no significant difference was found among the radii. In the Lowland site, no difference was found for the proportion of non-negligible results, either; there were, however, significantly fewer statistically significant results at the 9 m radius compared to the set of three other radii or to the 100 m radius (p<0.05).

## Discussion

On the Lowland site, estimators of ALS variables yielded mostly inconclusive results on the magnitude of their effect on floristic variables (60% “no-info”). On the Mountain site, even if estimators were inconclusive for nearly half of the models (44% “no-info”), the number of models with significant and non-negligible effects was significantly higher compared to Lowland site (27% and 8% respectively).

Several factors may explain the lower impact of forest structure observed in the Lowland site. Firstly, the low number of field plots in the Lowland site may have caused noisy responses when assessing the effect of some ALS variables, and this may explain the considerable number of inconclusive cases. Secondly, low variability in environmental variables might limit ecological responses at the Lowland site, where *Solrad*, *TTS* and *Slope*, which were assessed for all the field plots, had a lower standard deviation than on the Mountain site (Table 1).

We also observed an interesting inversion of the direction of non-negligible results between the Lowland site (only negative) and the Mountain site (mostly positive). Even after restricting the analysis on ALS variables related to volume and height, this result indicates that for the Mountain site most of the non-negligible effects were positive for species abundance in higher or “denser” stands. These results mainly concerned species abundance. It recalls the differences observed between lowland forests [50] and mountain forests [17] with a shift from mostly negative effects of cover to more mixed effects, including positive ones, depending on the ecological group and the ecological conditions involved. It remains to be seen if this inversion in the direction of effects can be generalized to other lowland and mountain forests.

The results obtained for the Mountain site were more diverse regarding the influence of each ALS variable. These results highlight the possibly significant effects of forest structure on ground-layer floristic biodiversity, in particular for the Mountain site with uneven-aged and partially mixed stands. However, previous studies predicting floristic biodiversity using models based on environmental and forest structure variables failed to detect any significant effects of forest structure variables in temperate forests [53] or oak forests [54], the latter focusing on pole stage oak forests. These differing conclusions could be linked to the relationship between biodiversity and forest structure as it might change according to abiotic environmental conditions [17]. Biotic conditions at the study site, i.e. the diversity of forest species and the range of forest structures, might also have an influence. Finally, the failure to detect any relationship in past studies might also be explained by the difficulty to measure relevant forest structure variables in the field. To date, most studies on floristic biodiversity have used field-measured variables, while most of the ALS-derived variables that we found to have significant non-negligible effects are arduous to measure in the field (for example, height measurements, maximum gap size or the attenuation rate *C*_*r*_ providing information on the quantity of light reaching the understory).

Some ALS variables had effects only on ecological group richness for the Mountain site. Two models for shade-tolerant group richness were negatively impacted by *Gap*_*max*_ and two models of heliophilous group richness by *H*_*median*_ and *Vol*_*can*_. One model of heliophilous group richness was positively impacted by cover rate (*C*_*r*_). The four negative effects can be directly explained by the light-preference trends of each group. Indeed, *H*_*median*_ and *Vol*_*can*_ were positively correlated to both cover fraction and cover rate for the Mountain site (ranging from 0.59 to 0.69 for the 15 m radius). *Gap*_*max*_ was negatively correlated to both cover fraction and cover rate. Richness values ranged from 0 to 2 for the shade-tolerant group (mean = 0.5 and standard deviation = 0.6) and from 0 to 20 for the heliophilous group (mean = 5.3 and standard deviation = 3.7). As a result, despite the positive impact of a high cover rate on the shade-tolerant group, global plant richness is likely to be low when the cover rate is high. High total canopy cover is known to significantly reduce plant diversity in beech stands [55]. For the Mountain site, which is dominated by European beech (*Fagus sylvatica* L.), the same trend is likely to be observed, though in this study we did not include total plant richness. Finally, the positive impact of cover rate (*C*_*r*_) on heliophilous group richness is more difficult to interpret with the available data.

Only two abundance models were highly improved by ALS variables for abundance in the Lowland site. Wood false brome (brsy—*Brachypodium sylvaticum* (Huds.) P.Beauv.), an intermediate-light species, and wood bluegrass (pone- *Poa nemoralis* L.), a heliophilous species, were both negatively sensitive to maximum tree height (*H*_*max*_). *H*_*max*_ was found to be slightly negatively correlated with both cover fraction and cover rate (e.g. -0.28 and -0.22, respectively, at the 15 m radius) and slightly positively correlated with *Gap*_*max*_ (e.g. 0.30 at the 15 m radius). For the Mountain site, abundance models of three intermediate-light species were greatly improved by ALS variables. Two models for wood sorrel were also improved with positive responses to canopy volume, and one European blueberry model showed a positive response to the coefficient of variation for leaf density. Red raspberry (Ruid—*Rubus idaeus* L.) abundance was significantly impacted with positive responses to all the ALS variables related to horizontal canopy distribution, to one height variable (*H*_*mean*_), and also to the *Gini* coefficient.

Impacts of forest structure on abundance indicators could have been significantly impacted in the same direction by ALS variables that are partially negatively correlated. For example, red raspberry abundance was positively impacted by *Gini* and *Gap*_*max*_, which are positively correlated (0.76 at the 15 m radius), but raspberry was also positively impacted by cover fraction (*Cf*) and cover rate (*C*_*r*_), which are both negatively correlated to the two previous variables. A single ALS variable might be insufficient to summarize vegetation structure, which is a complex environmental feature. A temporal dimension may also partially explain these apparent contradictions. The time interval between structure measurement and biodiversity observation is likely to be a source of additional noise.

The degree to which forest structure can be successfully characterized regarding a given issue often depends on scale [56]. In our study, there may have been an influence of the surrounding forest structure on species inside the field plots through processes such as edge effects and seed dispersal. Remote sensing enabled us to investigate the influence of forest structure on local biodiversity at different scales. The number of ALS variables found to have significant non-negligible effects, either positive or negative, on biodiversity indicators increased as the radius used to extract the variables increased, and this remained true up to 100 m, thereafter a decrease occurred (Fig 4). Therefore, characterizing the forest structure surrounding the field plots improved the ability of the model to link forest structure and local biodiversity indicators. We believe that this is a second improvement offered by ALS, since such information is very time consuming to measure in the field.

Several sources of error may have affected the accuracy of the species abundance and richness models in this study. Firstly, the field plots were not inventoried at the same time as the ALS acquisitions, but rather over a period of five years. A period of several years was necessary to carry out enough field inventories. Potential bias could be avoided by assigning a weight to each individual plot depending on the time interval between the field measurements and ALS data acquisition. Secondly, pulse density and plot positioning may affect ALS variable estimations to an extent that depends on stand type [57,58]. The ALS variables used, e.g. height variables (H_median_ and H_mean_), and variables derived from the raster canopy height model (Gap_max_ or C_f_), are likely to show low sensitivity to pulse density [59]. Positioning errors lead to discrepancies between the trees considered in a plot and those that are actually measured for ALS data, thus potentially reducing the capacity of ALS variables to reliably describe the vegetation structure within a given field plot. We expect a smaller impact of these discrepancies as the radius used to compute ALS variables increases. Plot size might partly explain that our tests were inconclusive for all but three models on the Lowland site when we considered only the variables computed with the same radius as the field plots, i.e. 9 m. Thirdly, the number of ALS variables tested in our study was limited since it was impossible to test several variables simultaneously in the Bayesian models we developed. Each test was also quite time-consuming. Wider ALS surveys with datasets more suited to Bayesian models would allow us to extend our approach. Furthermore, we recognize that our approach is limited in two respects. Firstly, for numerical reasons, we used a single value for abiotic variables, and did not include all the posterior distributions of the first model. Secondly, the biotic variable *C*_*tot*_ in our first-stage models was replaced by one of the ALS variables in the second-stage models, thus probably making these abiotic parameters sub-optimal; yet the variable *C*_*tot*_ was one of the dendrometric variables that was ecologically the closest to many of the ALS variables we used which were related to stand openness.

Floristic species abundance and richness are known to largely depend on forest structure and forest composition [37,60]. The scope of this study was limited to the impact of forest structure. Our approach would be further enhanced by combining ALS data sets with optical remote-sensing data sets [61]. This combination would not only provide information on the structural properties of the forest, but also on spectral properties linked to species composition [11]. We feel that integrating ALS variables within ecological research on floristic biodiversity in a wide range of forest types is a very promising step forward.

## Conclusion

Biodiversity monitoring and conservation are essential components of sustainable management in forest ecosystems, but they require progress in biodiversity modelling to facilitate the practical implementation of specific guidelines. Forest structure is known to play a major role in ecology by affecting e.g. micro-climate and habitat quality. In this study, we used a Bayesian approach, which is suitable for modelling the complex links between biodiversity and environmental conditions, in order to investigate the ability of ALS data to characterize forest structure and improve floristic biodiversity models. We assessed model improvements depending on: the study site, the biodiversity indicator predicted, the component of forest structure described by the ALS variables, and the radius used to estimate ALS variables. Floristic biodiversity was assessed in the field using abundance and richness indices. This study highlighted the relevance of ALS data in quantifying forest structural characteristics in order to monitor floristic biodiversity. Shade-tolerant and heliophilous species richness were impacted by indicators linked to light penetration through the vegetation cover, with a corresponding reverse trend observed for the two groups. Our results also highlight the importance of being able to measure vegetation structure over an area beyond the plot on which the biodiversity is observed; neighbouring structure helped us explain local biodiversity.

The use of ALS data resulted in several original findings; for example, shade-tolerant species might behave like forest interior species by avoiding the vicinity of large gaps. We also confirmed that the responses of biodiversity indicators to forest structure variables did vary between the study sites. Obtaining both the vertical and horizontal components of the structure are likely to be necessary when modelling biodiversity, as evidenced by situations where a biodiversity indicator was impacted in the same direction by two negatively correlated ALS variables. Bayesian approaches, which in some ways have proved well suited to biodiversity modelling, would probably benefit from the availability of ALS surveys over larger areas. Finally, our results did not reveal a widespread impact of ALS variables on all floristic species, but rather a kaleidoscope of relationships between structural variables and floristic biodiversity indicators.

## Appendix: Bayesian models and MCMC process used to fit the models

### Models

A set of variables was used in a linear combination denoted as η to model the mean (or related quantity) of the distribution used to model species abundance or species richness (see Zilliox and Gosselin [17]). This gave the following equation for η at plot i:

$$\begin{array}{ll} \eta_{i}= & \xi_{0}+\xi_{1}Reaction_{1i}+\;\xi_{2}Reaction_{2i}+\xi_{3}Reaction_{3i}+\xi_{4}SWC_{1i}+\xi_{5}SWC_{2i}+\xi_{6}SWC_{3i}\\ & +\xi_{7}Tmean_{1i}+\xi_{8}Tmean_{2i}+\xi_{9}Tmean_{3i}+\xi_{10}Solrad_{1i}+\xi_{11}Solrad_{2i}+\xi_{12}Solrad_{3i}\\ & +\xi_{13}(1-TTS_{0i})TTS_{1i}+\xi_{14}(1-TTS_{0i})TTS_{2i}+\xi_{15}(1-TTS_{0i})TTS_{3i}+\xi_{16}TTS_{0i}\\ & +\xi_{17}\text{sin}\left(\text{min}\left(Slope_{i},\frac{\pi }{4}\right)\right)\text{cos}\left(Aspect_{i}\right)+\xi_{18}Indicator_{i} \end{array}$$

where the three vectors associated with Reaction, SWC, Tmean, and Solrad are the three components generated from a restricted cubic spline applied to the scaled variable with four default knots [16]; the three vectors associated with TTS are the three components generated from a restricted cubic spline, with 4 knots at 2.5, 4.5, 5.5 and 7 respectively, applied to the categorical variable transformed into a numerical variable; and the vector associated with Indicator is the dendrometric indicator used—here Ctot. For species richness, η was the logarithm of the mean of the count data distribution while in the case of species abundance, it was the logit function of the global mean of the latent variable used to model the probability of Braun-Blanquet classes. The probability distributions of the observed data were respectively the Bernoulli/Double Polya mixture-Poisson-Negative Binomial family for species richness—which allows for both under- and over-dispersion relative to the Poisson distribution [17]–and the MTUnlimited 2 zero-inflated cumulative beta distribution for abundance data. The priors for the ecological main effects were a weakly informative normal distribution with mean 0 and standard deviation 2. Other priors were also set to be weakly informative. Examples of the R-codes used to calculate the log-posterior density for abundance models can be found in the last supplementary file at: http://www.sciencedirect.com/science/article/pii/S1574954114001629#MMCvFirst.

### MCMC process

MCMC processThe Bayesian models were fitted through an adaptive Markov-Chain Monte Carlo (MCMC) process programmed in R and C, involving some C functions coded by F. Gosselin. This MCMC was inspired from Gregory [48]. To better treat the cases where statistical parameters are correlated, the algorithm developed by Gregory [48] mixes a parallel tempering algorithm—thus allowing swaps of states between trajectories of different temperatures, and a better exploration of a potentially multimodal log posterior distribution—and a differential evolution algorithm coupled with a Metropolis algorithm.

Four main modifications were made to the process described by Gregory [48]:

1. the classical parallel tempering algorithm was replaced by Baragatti’s [62] equi-energy moves parallel tempering algorithm;
2. Some elements from the differential evolution algorithm proposed by Vrugt et al. [63], such as variable crossover probabilities of (1/3; 2/3 and 1), were integrated into the process;
3. the Metropolis algorithm in Gregory [48] was replaced by a component-wise Metropolis-within-Gibbs algorithm, which uses a Gaussian random walk,
4. finally, an adaptive tuning of the differential evolution and Metropolis-within-Gibbs algorithms based on the diminishing adaptation condition [64] was included.

We considered 17 trajectories, four of which included temperature. We used Gelman-Rubin Rhat metrics to diagnose convergence of the MCMC but with a lower limit value than in Gelman et al. [46]—1.007 instead of 1.2. At convergence, the level of thinning was changed to reach a 0.01 average level of correlation of successive MCMC states.

## Supporting information

S1 Table Complete results for Lowland site models. Statistical indicators, i.e. *ΔDIC*, direction and magnitude, corresponding to each abundance or richness model with an ALS variable for the Lowland site.
(DOCX)

S2 Table Complete results for Mountain site models. Statistical indicators, i.e. *ΔDIC*, direction and magnitude, corresponding to each abundance or richness model with an ALS variable for the Mountain site.
(DOCX)
