# Supplementary material for: Use of airborne lidar data to improve plant species richness and diversity monitoring in lowland and mountain forests
Source: PLoS One. 2017 Sep 13;12(9):e0184524. doi: 10.1371/journal.pone.0184524 (PMC5597197; doi:10.1371/journal.pone.0184524)
Supplement: S1 Table — Statistical indicators, i.e. ΔDIC, direction and magnitude, corresponding to each abundance or richness model with an ALS variable for the Lowland site. (DOCX) [file pone.0184524.s001.docx]

**S1 Table.** Statistical indicators, i.e. *ΔDIC*, direction and magnitude, corresponding to each abundance or richness model with a lidar variable for the Lowland site.

|  |  | brsy (4) | | | casy (5) | | | gaod (3) | | | hehe (3) | | | laga (4) | | | mief (5) | | | anne (4) | | | pone (7) | | | scia | | | inter | | | helio | | |
| --- | --- | --- | --- | --- | --- | --- | --- | --- | --- | --- | --- | --- | --- | --- | --- | --- | --- | --- | --- | --- | --- | --- | --- | --- | --- | --- | --- | --- | --- | --- | --- | --- | --- | --- |
|  |  | ΔDIC | Dir | Mag | ΔDIC | Dir | Mag | ΔDIC | Dir | Mag | ΔDIC | Dir | Mag | ΔDIC | Dir | Mag | ΔDIC | Dir | Mag | ΔDIC | Dir | Mag | ΔDIC | Dir | Mag | ΔDIC | Dir | Mag | ΔDIC | Dir | Mag | ΔDIC | Dir | Mag |
| 9 m | *H_max_* | 1.85 |  |  | 1.54 |  |  | 1.410 |  |  | -1.350 | 0 |  | 1.770 |  |  | 1.950 |  |  | 0.730 |  |  | 1.760 |  |  | 2.25 |  | * | -3.89 | 0 |  | -1.86 | 0 |  |
|  | *H_median_* | 1.32 |  |  | 1.86 | 0 |  | 0.284 |  |  | 0.820 | 0 |  | 0.738 |  |  | 1.968 |  |  | 1.153 |  |  | -2.713 |  |  | -0.86 |  |  | 0.59 | 0 |  | 2.03 | 0 |  |
|  | *H_mean_* | 1.86 |  |  | 1.71 | 0 |  | -2.300 |  |  | 0.230 | 0 |  | -1.680 |  |  | 1.210 |  |  | 1.470 |  |  | 1.410 |  | * | 1.03 |  |  | 1.58 | 0 |  | 1.24 | 0 |  |
|  | $\sigma_{H}^{2}$ | -3.11 |  |  | -3.98 | 0 |  | 1.690 | 0 |  | -1.830 | 0 |  | 1.470 | 0 |  | 1.730 |  |  | 1.400 |  |  | 2.350 |  |  | 1.92 | 0 |  | -3.21 | 0 |  | -4.66 | 0 |  |
|  | *Gini* | -1.21 |  |  | -0.91 |  |  | -1.320 | 0 |  | 1.470 | 0 |  | -2.990 | 0 |  | 0.890 |  |  | 3.660 |  |  | 0.560 |  |  | 1.48 | 0 |  | -0.25 | 0 |  | -0.82 | 0 |  |
|  | *Cv_LAD_* | 3.02 |  |  | 1.59 |  |  | 0.810 |  |  | 2.010 | 0 |  | -0.540 |  |  | -2.340 |  |  | 1.390 |  |  | 0.390 |  |  | 2.04 | 0 |  | 1.96 | 0 |  | 2.15 | 0 |  |
|  | *Gap_max_* | 0.22 |  |  | 0.62 | 0 |  | 1.271 |  |  | 1.541 | 0 |  | 1.539 | 0 |  | 1.227 |  |  | 1.590 |  |  | 0.727 |  |  | 1.90 | 0 |  | 1.16 | 0 |  | -0.29 | 0 |  |
|  | *C_f_* | -0.20 |  |  | 0.17 |  |  | 1.657 |  |  | 1.818 | 0 |  | 1.839 | 0 |  | 1.856 |  |  | 1.609 |  |  | 1.625 |  |  | 2.13 | 0 |  | 1.02 | 0 |  | -0.76 | 0 |  |
|  | *C_r_* | -0.52 |  |  | 0.73 |  |  | 0.130 |  |  | 1.850 | 0 |  | -0.940 | 0 |  | 0.370 |  |  | 2.540 |  |  | 0.060 |  |  | 2.12 | 0 |  | 0.02 | 0 |  | -0.15 | 0 |  |
|  | *Vol_can_* | 1.22 |  |  | 1.79 | 0 |  | 0.350 |  |  | 0.682 | 0 |  | 0.590 |  |  | 2.018 |  |  | 0.996 |  |  | 0.655 |  | * | -1.24 |  |  | 0.52 | 0 |  | 2.12 | 0 |  |
| 50 m | *H_max_* | 1.62 |  | * | 1.18 |  |  | 1.440 |  |  | -1.500 |  | * | 1.600 |  |  | 1.330 |  |  | -1.100 |  |  | 1.750 |  |  | 1.45 |  |  | 2.04 |  | * | -0.65 |  |  |
|  | *H_median_* | 1.23 |  |  | 1.38 |  |  | -0.422 |  |  | 1.095 | 0 |  | 0.565 |  |  | 1.773 |  |  | 1.281 |  |  | -3.972 |  | * | -3.06 |  | * | 0.76 | 0 |  | 1.66 | 0 |  |
|  | *H_mean_* | 1.81 |  |  | 1.83 | 0 |  | -0.210 |  |  | -0.390 | 0 |  | -1.590 |  |  | 1.740 |  |  | 1.390 |  |  | 1.520 |  | * | 1.60 |  | * | 1.31 | 0 |  | 0.76 | 0 |  |
|  | $\sigma_{H}^{2}$ | -2.90 |  |  | -0.44 |  |  | 1.640 | 0 |  | -0.870 | 0 |  | 1.860 | 0 |  | 1.430 |  |  | 1.180 |  |  | 1.680 |  |  | 1.67 | 0 |  | -4.26 | 0 |  | -3.77 | 0 |  |
|  | *Gini* | -2.40 |  |  | -1.57 |  |  | -0.600 |  |  | 2.010 | 0 |  | -3.110 | 0 |  | 1.190 |  |  | 1.710 |  |  | 0.750 |  |  | 0.44 | 0 |  | -0.13 | 0 |  | -0.49 | 0 |  |
|  | *Cv_LAD_* | -0.71 |  |  | 1.30 |  |  | 0.916 |  |  | -2.471 |  | * | 1.646 |  |  | 1.390 |  |  | 1.620 |  |  | -0.240 |  |  | 0.15 |  |  | -1.17 | 0 |  | -2.70 |  | * |
|  | *Gap_max_* | 0.61 |  |  | 1.59 | 0 |  | 1.552 |  |  | 1.159 | 0 |  | 1.218 |  |  | 0.960 |  |  | 1.890 |  |  | 2.209 |  |  | 1.90 | 0 |  | 2.13 | 0 |  | 0.24 | 0 |  |
|  | *C_f_* | -2.57 |  | * | 1.20 |  |  | 1.150 | 0 |  | 1.850 | 0 |  | 0.740 |  |  | -0.210 |  |  | 1.850 |  |  | -0.540 |  |  | 1.88 | 0 |  | -0.62 | 0 |  | 0.55 | 0 |  |
|  | *C_r_* | -2.18 |  |  | 1.51 | 0 |  | 0.140 |  |  | 2.080 | 0 |  | -0.070 | 0 |  | -0.630 |  |  | 1.640 |  |  | -1.370 |  |  | 2.10 | 0 |  | -0.24 | 0 |  | 0.47 | 0 |  |
|  | *Vol_can_* | 1.20 |  |  | 1.65 |  |  | -0.652 |  |  | 1.018 | 0 |  | 0.879 |  |  | 1.815 |  |  | 0.873 |  |  | -4.177 |  | * | -3.32 |  | * | 1.16 | 0 |  | 2.00 | 0 |  |
| 100 m | *H_max_* | 1.72 | - | * | 1.00 |  |  | 1.490 |  |  | -0.120 |  | * | 1.370 |  |  | 1.290 |  |  | -2.140 |  |  | 1.770 | -- | ** | 1.47 |  |  | 1.75 |  | * | -1.26 |  |  |
|  | *H_median_* | 1.45 |  |  | 1.56 |  |  | -0.106 |  |  | 0.926 | 0 |  | 0.978 |  |  | 1.518 |  |  | 1.041 |  |  | -1.723 |  | * | -2.68 |  | * | 0.27 | 0 |  | 2.15 | 0 |  |
|  | *H_mean_* | 1.50 |  |  | 1.78 | 0 |  | -0.640 |  |  | -0.500 | 0 |  | -1.650 |  |  | 1.520 |  |  | 1.750 |  |  | 2.020 |  | * | 1.97 |  | * | 1.87 | 0 |  | 1.56 | 0 |  |
|  | $\sigma_{H}^{2}$ | 0.90 |  |  | 1.85 | 0 |  | 1.530 | 0 |  | 1.260 | 0 |  | 1.710 | 0 |  | 0.890 |  |  | 2.020 |  |  | 1.090 |  |  | 1.85 | 0 |  | -1.72 | 0 |  | 0.40 | 0 |  |
|  | *Gini* | -0.67 |  | * | 0.68 |  |  | -1.520 |  |  | 1.970 | 0 |  | -2.100 | 0 |  | 1.730 |  |  | 1.590 |  |  | -0.600 |  |  | -0.32 | 0 |  | 0.71 | 0 |  | 1.09 | 0 |  |
|  | *Cv_LAD_* | 1.64 |  |  | 0.34 |  |  | 1.760 |  |  | 0.770 |  | * | 1.820 |  |  | 2.370 |  |  | -0.430 |  |  | 4.970 |  |  | 2.26 |  |  | 1.98 | 0 |  | -2.45 |  | * |
|  | *Gap_max_* | -0.91 |  |  | 1.36 |  |  | -4.772 |  | * | 2.145 | 0 |  | 1.445 |  |  | 1.688 |  |  | 2.413 |  |  | 2.062 |  |  | 1.52 | 0 |  | 0.56 | 0 |  | 0.73 | 0 |  |
|  | *C_f_* | -0.83 |  |  | 1.84 |  |  | 1.210 |  |  | 1.850 | 0 |  | 1.240 |  |  | 1.570 |  |  | 1.470 |  |  | 0.470 |  |  | -1.00 | 0 |  | 1.47 | 0 |  | 1.82 | 0 |  |
|  | *C_r_* | -0.31 |  | * | 1.76 | 0 |  | 0.470 | 0 |  | 2.050 | 0 |  | 0.650 | 0 |  | 0.830 |  |  | 1.550 |  |  | -0.850 |  |  | 0.99 | 0 |  | 1.70 | 0 |  | 1.78 | 0 |  |
|  | *Vol_can_* | 0.73 |  |  | 1.77 |  |  | -0.158 |  |  | 1.253 | 0 |  | 1.072 |  |  | 1.122 |  |  | 0.738 |  |  | -0.290 |  | * | -2.48 |  | * | 0.38 | 0 |  | 2.03 | 0 |  |
| 200 m | *H_max_* | 1.50 |  |  | 0.18 | 0 |  | 1.320 | 0 |  | 1.120 | 0 |  | 1.660 |  |  | 1.430 |  |  | 1.960 |  |  | 1.800 | - | ** | 1.94 | 0 |  | 1.77 | 0 |  | -4.89 | 0 |  |
|  | *H_median_* | 2.47 |  |  | 1.81 | 0 |  | -4.575 |  | * | 0.674 | 0 |  | -0.266 |  |  | -0.742 |  |  | 1.028 |  |  | 3.081 |  |  | -3.30 |  | * | 1.38 | 0 |  | 1.88 | 0 |  |
|  | *H_mean_* | 1.70 |  |  | 1.83 | 0 |  | 0.340 |  | * | -2.800 | 0 |  | -0.330 |  |  | 1.990 |  |  | 1.000 |  |  | 2.510 |  |  | 2.20 |  | * | 1.24 | 0 |  | 2.05 | 0 |  |
|  | $\sigma_{H}^{2}$ | 0.95 |  |  | 1.92 | 0 |  | 1.940 |  |  | 1.800 | 0 |  | 1.650 | 0 |  | 1.190 |  |  | 1.800 |  |  | 0.960 |  |  | 1.99 | 0 |  | -1.64 | 0 |  | 0.69 | 0 |  |
|  | *Gini* | 1.69 |  |  | 1.56 |  |  | -0.330 |  |  | 2.070 | 0 |  | 0.890 | 0 |  | 2.160 |  |  | -0.100 |  |  | 1.820 |  |  | 1.94 | 0 |  | 2.31 | 0 |  | 1.93 | 0 |  |
|  | *Cv_LAD_* | 1.65 |  |  | -0.01 |  |  | 1.610 |  |  | 1.640 |  |  | 1.660 | 0 |  | 2.120 |  |  | 2.010 |  |  | 1.610 |  |  | 2.37 | 0 |  | 1.85 | 0 |  | -4.97 | 0 |  |
|  | *Gap_max_* | 2.48 |  |  | 1.79 | 0 |  | 0.731 |  |  | 1.894 | 0 |  | 1.443 |  |  | 0.599 |  |  | 0.348 |  |  | 2.813 |  |  | 2.19 | 0 |  | 1.15 | 0 |  | -1.07 | 0 |  |
|  | *C_f_* | 1.57 |  |  | 1.88 |  |  | 1.680 |  |  | 1.120 | 0 |  | 1.570 |  |  | 1.960 |  |  | -1.490 |  |  | 0.640 |  |  | 2.01 | 0 |  | 1.94 | 0 |  | 1.01 | 0 |  |
|  | *C_r_* | 1.54 |  |  | 1.88 |  |  | 1.540 | 0 |  | 1.420 | 0 |  | 1.640 | 0 |  | 1.650 |  |  | -0.660 |  |  | 1.420 |  |  | 2.01 | 0 |  | 1.70 | 0 |  | 1.21 | 0 |  |
|  | *Vol_can_* | 1.54 |  |  | 1.98 | 0 |  | -0.718 |  |  | 1.797 | 0 |  | 1.231 |  |  | -0.179 |  |  | 1.043 |  |  | 0.326 |  |  | -2.53 |  | * | 1.07 | 0 |  | 1.80 | 0 |  |
