# Supplementary material for: Use of airborne lidar data to improve plant species richness and diversity monitoring in lowland and mountain forests
Source: PLoS One. 2017 Sep 13;12(9):e0184524. doi: 10.1371/journal.pone.0184524 (PMC5597197; doi:10.1371/journal.pone.0184524)
Supplement: S2 Table — Statistical indicators, i.e. ΔDIC, direction and magnitude, corresponding to each abundance or richness model with an ALS variable for the Mountain site. (DOCX) [file pone.0184524.s002.docx]

**S2 Table.** Statistical indicators, i.e. *ΔDIC*, direction and magnitude, corresponding to each abundance or richness model with a lidar variable for the Mountain site.

|  |  | capi (5) | | | delf (8) | | | hehe (3) | | | oxac (4) | | | ruid (5) | | | vamy (5) | | | dipu (5) | | | atfi (3) | | | scia | | | inter | | | helio | | |
| --- | --- | --- | --- | --- | --- | --- | --- | --- | --- | --- | --- | --- | --- | --- | --- | --- | --- | --- | --- | --- | --- | --- | --- | --- | --- | --- | --- | --- | --- | --- | --- | --- | --- | --- |
|  |  | ΔDIC | Dir | Mag | ΔDIC | Dir | Mag | ΔDIC | Dir | Mag | ΔDIC | Dir | Mag | ΔDIC | Dir | Mag | ΔDIC | Dir | Mag | ΔDIC | Dir | Mag | ΔDIC | Dir | Mag | ΔDIC | Dir | Mag | ΔDIC | Dir | Mag | ΔDIC | Dir | Mag |
| 15 m | *H_max_* | 0.95 |  |  | -3.04 |  | * | 1.54 | 0 |  | 1.64 | 0 |  | 0.76 |  |  | 2.01 | 0 |  | 1.46 |  |  | 1.05 |  |  | 1.61 |  |  | 0.93 |  |  | 2.16 | 0 |  |
|  | *H_median_* | 0.85 |  |  | 0.88 | 0 |  | -3.19 |  | * | -7.08 |  | ** | -0.14 |  |  | 2.14 | 0 |  | -5.96 |  | ** | 0.13 | 0 |  | -0.06 |  |  | 1.90 | 0 |  | -12.52 | - | ** |
|  | *H_mean_* | -3.96 |  | * | 0.53 | 0 |  | 1.34 | 0 |  | -10.75 |  | ** | 1.49 | 0 |  | 0.48 |  |  | -6.01 |  | ** | 2.01 | 0 |  | 0.82 |  |  | 0.93 | 0 |  | -6.09 |  | ** |
|  | $\sigma_{H}^{2}$ | 1.79 |  |  | -1.47 |  | * | 0.45 | 0 |  | 1.29 | 0 |  | 2.20 |  |  | 0.79 |  |  | 1.20 |  |  | 2.59 | 0 |  | 1.73 |  |  | 1.82 | 0 |  | 2.17 | 0 |  |
|  | *Gini* | 1.79 | 0 |  | 1.98 | 0 |  | 1.08 | 0 |  | -0.11 | 0 |  | -19.67 | + | ** | 1.66 | 0 |  | 1.30 | 0 |  | 0.26 | 0 |  | 1.16 |  |  | 0.48 | 0 |  | -7.12 | 0 | ** |
|  | *Cv_LAD_* | -0.19 |  |  | -4.63 |  | ** | 2.36 | 0 |  | 1.63 | 0 |  | -0.17 |  |  | 1.95 | 0 |  | 2.17 |  |  | -0.56 |  |  | 0.35 |  |  | -1.83 |  | * | -0.68 |  | * |
|  | *Gap_max_* | 1.63 | 0 |  | -5.67 |  | ** | -2.50 |  | * | 0.62 | 0 |  | -26.38 | ++ | ** | 0.01 |  |  | 0.14 | 0 |  | 0.23 | 0 |  | 0.31 |  |  | 1.82 | 0 |  | -5.91 | 0 | ** |
|  | *C_f_* | 1.40 | 0 |  | 1.44 | 0 |  | 2.13 | 0 |  | 0.32 | 0 |  | -19.23 | ++ | ** | 1.60 | 0 |  | 1.98 | 0 |  | -0.31 | 0 |  | 0.23 |  |  | 0.62 | 0 |  | -8.86 |  | ** |
|  | *C_r_* | 0.65 |  |  | -2.42 |  | * | 1.48 | 0 |  | -0.25 | 0 |  | -18.89 | + | ** | 0.27 |  |  | 1.90 | 0 |  | -0.22 | 0 |  | -0.73 |  |  | 0.87 | 0 |  | -9.60 |  | ** |
|  | *Vol_can_* | 1.37 |  |  | 1.90 | 0 |  | 1.13 |  |  | -5.15 |  | ** | -1.61 |  |  | 1.49 |  |  | -3.08 |  | * | -0.17 | 0 |  | 1.14 |  |  | 1.68 | 0 |  | -10.54 |  | ** |
| 50 m | *H_max_* | 1.78 |  |  | 1.04 | 0 |  | -0.35 |  |  | 1.46 | 0 |  | 1.45 |  |  | 1.31 |  |  | 0.60 | 0 |  | 2.11 |  |  | 1.19 |  |  | 1.51 | 0 |  | 2.04 | 0 |  |
|  | *H_median_* | 1.49 | 0 |  | 1.52 | 0 |  | -6.28 |  | ** | -9.34 |  | ** | -0.59 |  |  | 1.80 | 0 |  | -6.68 |  | ** | 1.48 | 0 |  | -0.98 |  |  | 1.48 | 0 |  | -10.99 |  | ** |
|  | *H_mean_* | -2.88 |  | * | 1.04 | 0 |  | 0.61 |  |  | -13.86 | + | ** | 1.85 | 0 |  | 0.02 |  |  | -7.29 |  | ** | 1.84 | 0 |  | 0.54 |  |  | 0.87 | 0 |  | -5.41 |  | ** |
|  | $\sigma_{H}^{2}$ | 2.01 |  |  | -4.25 |  | ** | 2.31 | 0 |  | 1.29 | 0 |  | 2.01 | 0 |  | -0.20 |  |  | 1.40 | 0 |  | 1.46 | 0 |  | 1.62 |  |  | 1.61 | 0 |  | 2.06 | 0 |  |
|  | *Gini* | 1.67 | 0 |  | 2.06 | 0 |  | -2.16 |  | * | -1.65 |  |  | -13.35 | + | ** | 1.60 | 0 |  | -0.60 |  |  | 1.49 | 0 |  | -0.09 |  |  | 0.08 | 0 |  | -11.17 |  | ** |
|  | *Cv_LAD_* | 1.33 |  |  | 1.87 | 0 |  | -2.69 |  | * | 1.43 | 0 |  | 1.37 |  |  | 2.02 |  |  | -0.63 | 0 |  | 1.51 |  |  | 1.36 |  |  | 1.51 | 0 |  | 1.95 | 0 |  |
|  | *Gap_max_* | 1.86 | 0 |  | -4.99 |  | ** | 2.14 | 0 |  | -3.44 |  | * | -19.79 | + | ** | 0.48 | 0 |  | 1.90 | 0 |  | 1.56 | 0 |  | -2.86 |  | * | 0.97 | 0 |  | -12.21 |  | ** |
|  | *C_f_* | 1.90 | 0 |  | 1.82 | 0 |  | 0.05 |  |  | -0.77 |  |  | -17.02 | + | ** | 2.03 | 0 |  | 1.59 | 0 |  | 1.67 | 0 |  | -0.99 |  |  | 0.06 | 0 |  | -11.79 |  | ** |
|  | *C_r_* | 1.31 | 0 |  | 0.65 | 0 |  | -2.19 |  | * | -1.34 |  |  | -17.45 | + | ** | 2.03 | 0 |  | 1.67 | 0 |  | 1.53 | 0 |  | -3.10 |  | * | -0.54 | 0 |  | -13.10 | + | ** |
|  | *Vol_can_* | 1.41 |  |  | 1.94 | 0 |  | -1.09 |  |  | -10.37 |  | ** | -2.04 |  |  | 2.07 | 0 |  | -5.04 |  | ** | 1.83 | 0 |  | 0.67 |  |  | 1.93 | 0 |  | -11.57 |  | ** |
| 100 m | *H_max_* | 0.64 |  |  | 1.51 |  |  | 1.60 | 0 |  | 1.78 | 0 |  | 0.22 |  |  | -5.92 | + | ** | 1.11 | 0 |  | -0.95 |  |  | 1.87 | 0 |  | 1.55 | 0 |  | 1.55 | 0 |  |
|  | *H_median_* | 1.53 | 0 |  | 0.49 |  |  | -7.83 |  | ** | -10.80 | + | ** | -1.62 |  |  | 0.08 |  |  | -1.12 |  |  | 1.91 | 0 |  | -1.42 |  |  | 0.62 | 0 |  | -9.47 |  | ** |
|  | *H_mean_* | -0.68 |  |  | 1.57 | 0 |  | -0.63 |  |  | -14.50 | + | ** | 1.61 | 0 |  | 0.56 |  |  | -5.86 |  | ** | 2.12 | 0 |  | 0.81 |  |  | 1.30 | 0 |  | -6.40 |  | ** |
|  | $\sigma_{H}^{2}$ | 0.50 |  |  | -2.20 |  | * | 1.52 | 0 |  | -1.00 | 0 |  | 1.73 | 0 |  | -1.55 |  |  | 1.30 | 0 |  | 1.98 | 0 |  | 1.53 | 0 |  | 0.24 | 0 |  | 2.03 | 0 |  |
|  | *Gini* | 1.68 | 0 |  | 2.05 | 0 |  | -4.11 |  | * | -3.80 |  | * | -8.91 | + | ** | 0.94 |  |  | 0.56 | 0 |  | 1.80 | 0 |  | -1.07 |  |  | -0.38 | 0 |  | -11.38 |  | ** |
|  | *Cv_LAD_* | 0.84 |  |  | 1.98 | 0 |  | 2.23 | 0 |  | 1.90 | 0 |  | 0.23 |  |  | -5.40 | + | ** | 0.61 | 0 |  | -1.46 |  |  | 1.60 | 0 |  | 1.71 | 0 |  | 1.94 | 0 |  |
|  | *Gap_max_* | 1.57 | 0 |  | -6.17 |  | ** | 1.36 | 0 |  | -5.67 |  | ** | -11.37 | + | ** | 1.67 | 0 |  | 2.06 | 0 |  | 2.26 | 0 |  | -6.90 | - | ** | 0.36 | 0 |  | -12.13 |  | ** |
|  | *C_f_* | 1.71 | 0 |  | 1.78 | 0 |  | -1.84 |  | * | -2.59 |  | * | -11.09 | + | ** | 1.72 |  |  | 1.68 | 0 |  | 1.90 | 0 |  | -1.90 |  |  | -0.05 | 0 |  | -10.65 |  | ** |
|  | *C_r_* | 1.71 | 0 |  | -0.35 |  |  | -3.40 |  | * | -2.75 |  | * | -11.65 | + | ** | 1.92 | 0 |  | 1.83 | 0 |  | 1.84 | 0 |  | -3.95 |  | * | -0.39 | 0 |  | -10.46 |  | ** |
|  | *Vol_can_* | 1.34 |  |  | 1.94 | 0 |  | -3.24 |  | * | -11.67 | + | ** | -0.91 |  |  | 2.00 | 0 |  | -3.02 |  | * | 1.82 | 0 |  | 0.84 |  |  | 1.67 | 0 |  | -12.53 | - | ** |
| 200 m | *H_max_* | 1.98 | 0 |  | 0.78 |  |  | 0.95 | 0 |  | 0.52 | 0 |  | -1.67 |  |  | -7.15 | + | ** | 1.46 | 0 |  | 0.63 |  |  | 1.51 | 0 |  | 1.93 | 0 |  | 1.77 | 0 |  |
|  | *H_median_* | 1.55 |  |  | 1.07 | 0 |  | -8.33 |  | ** | -14.43 | + | ** | -0.15 |  |  | -4.36 |  | ** | 0.73 | 0 |  | 1.41 | 0 |  | -3.40 |  | * | 1.36 | 0 |  | -5.77 |  | ** |
|  | *H_mean_* | -0.92 |  |  | 1.58 | 0 |  | -1.87 |  | * | -16.14 | + | ** | 1.27 | 0 |  | 0.25 |  |  | -3.55 |  | * | 2.03 | 0 |  | 0.51 |  |  | 0.87 | 0 |  | -4.88 |  | ** |
|  | $\sigma_{H}^{2}$ | 1.61 | 0 |  | -0.04 | 0 |  | 1.72 | 0 |  | 1.33 | 0 |  | 1.77 | 0 |  | -1.62 |  |  | 1.68 | 0 |  | 2.01 | 0 |  | 1.48 | 0 |  | 2.04 | 0 |  | 1.97 | 0 |  |
|  | *Gini* | 1.94 | 0 |  | 1.45 | 0 |  | -5.33 |  | ** | -5.36 |  | ** | -2.28 |  |  | -3.45 |  | * | 0.45 | 0 |  | 2.22 | 0 |  | -2.55 |  | * | 1.32 | 0 |  | -5.53 |  | ** |
|  | *Cv_LAD_* | 1.95 | 0 |  | 1.15 | 0 |  | 1.76 | 0 |  | 0.50 | 0 |  | -2.40 |  | * | -5.73 |  | ** | 1.82 | 0 |  | 0.36 |  |  | 1.76 | 0 |  | 2.01 | 0 |  | 1.98 | 0 |  |
|  | *Gap_max_* | 1.76 | 0 |  | -5.75 |  | ** | 0.38 | 0 |  | -5.68 |  | ** | -3.72 |  | * | 2.00 | 0 |  | 1.12 |  |  | 1.63 | 0 |  | -5.92 | - | ** | 2.21 | 0 |  | -2.40 | 0 | * |
|  | *C_f_* | 1.56 | 0 |  | 2.08 | 0 |  | -2.96 |  | * | -3.01 |  | * | -4.07 |  | * | -0.90 |  |  | 1.33 | 0 |  | 2.18 | 0 |  | -2.59 |  | * | 1.06 | 0 |  | -5.38 | 0 | ** |
|  | *C_r_* | 1.49 | 0 |  | 0.64 | 0 |  | -4.54 |  | ** | -2.82 |  | * | -4.75 |  | * | 0.23 |  |  | 1.09 | 0 |  | 1.85 | 0 |  | -3.98 |  | * | 0.67 | 0 |  | -5.14 | 0 | ** |
|  | *Vol_can_* | 1.79 | 0 |  | 1.65 | 0 |  | -4.24 |  | * | -12.60 | + | ** | 0.71 | 0 |  | 1.61 |  |  | -1.83 |  |  | 1.89 | 0 |  | 1.21 |  |  | 1.83 | 0 |  | -8.05 |  | ** |
